# The Influence of MMA Esterification on Interfacial Adhesion and Mechanical Properties of Hybrid Kenaf Bast/Glass Fiber Reinforced Unsaturated Polyester Composites

**DOI:** 10.3390/ma14092276

**Published:** 2021-04-28

**Authors:** Rozyanty Rahman, Syed Zhafer Firdaus Syed Putra, Shayfull Zamree Abd Rahim, Irwana Nainggolan, Bartłomiej Jeż, Marcin Nabiałek, Luqman Musa, Andrei Victor Sandu, Petrica Vizureanu, Mohd Mustafa Al Bakri Abdullah, Dariusz Kwiatkowski, Izabela Wnuk

**Affiliations:** 1 Faculty of Chemical Engineering Technology, Universiti Malaysia Perlis, Perlis 01000, Malaysia; syedzhafer@gmail.com (S.Z.F.S.P.); shayfull@unimap.edu.my (S.Z.A.R.); luqman@unimap.edu.my (L.M.); mustafa_albakri@unimap.edu.my (M.M.A.B.A.); 2 Center of Excellence Geopolymer and Green Technology (CEGeoGTech), Universiti Malaysia Perlis, Perlis 01000, Malaysia; sav@tuiasi.ro (A.V.S.); peviz@tuiasi.ro (P.V.); 3 Faculty of Mechanical Engineering Technology, Universiti Malaysia Perlis, Perlis 01000, Malaysia; 4 Department of Chemistry, Faculty of Mathematic and Natural Sciences, Universitas Sumatera Utara, Medan 20155, Indonesia; irwana@usu.ac.id; 5 Department of Physics, Częstochowa University of Technology, 42-200 Częstochowa, Poland; bartek199.91@o2.pl (B.J.); nmarcell@wp.pl (M.N.); izabela.wnuk@pcz.pl (I.W.); 6 Faculty of Material Science and Engineering, Gheorghe Asachi Technical University of Iasi, 41 D. Mangeron St., 700050 Iasi, Romania; 7 National Institute for Research and Development for Environmental Protection INCDPM, 294 SplaiulInde-Pendentei, 060031 Bucharest, Romania; 8 Faculty of Mechanical Engineering and Computer Science, Częstochowa University of Technology, 42-200 Częstochowa, Poland; kwiatkowski@ipp.pcz.pl

**Keywords:** natural fiber, kenaf fiber, MMA-treatment, hybrid composite, mechanical properties

## Abstract

The demand for natural fiber hybrid composites for various applications has increased, which is leading to more research being conducted on natural fiber hybrid composites due to their promising mechanical properties. However, the incompatibility of natural fiber with polymer matrix limits the performance of the natural fiber hybrid composite. In this research work, the mechanical properties and fiber-to-matrix interfacial adhesion were investigated. The efficiency of methyl methacrylate (MMA)-esterification treatments on composites’ final product performance was determined. The composite was prepared using the hand lay-up method with varying kenaf bast fiber (KBF) contents of 10, 15, 20, 25, 30, 35 (weight%) and hybridized with glass fiber (GF) at 5 and 10 (weight%). Unsaturated polyester (UPE) resin and methyl ethyl ketone peroxide (MEKP) were used as binders and catalysts, respectively. Scanning electron microscopy (SEM) and Fourier-transform infrared spectroscopy (FTIR) were used to examine the effects of MMA-esterification treatment on tensile strength and morphology (tensile fracture and characterization of MMA-esterification treatment) of the composite fabricated. The tensile strength of MMA-treated reinforced UPE and hybrid composites are higher than that of untreated composites. As for MMA treatment, 90 min of treatment showed the highest weight percent gain (WPG) and tensile strength of KBF-reinforced UPE composites. It can be concluded that the esterification of MMA on the KBF can lead to better mechanical properties and adhesion between the KFB and the UPE matrix. This research provides a clear reference for developing hybrid natural fibers, thus contributing to the current field of knowledge related to GF composites, specifically in transportation diligences due to their properties of being lightweight, superior, and involving low production cost.

## 1. Introduction

Hybrid fiber composites are materials that consist of two or more fiber reinforcements in a composite system. Combining these reinforcements into the polymer matrix procedure is known as a hybridization process [1,2]. The matrix serves as a binder and gives extra strength to the composite [3]. Fiber is an important and primary part of the composites industry. In fiber-reinforced plastic (FRP) composites, fiber serves as reinforcements by providing strength and stiffness to the structure, while polymer matrices act as the binder to hold the fiber in place [4,5,6]. Various inorganic and organic fibers, such as glass, carbon, graphite, aramid, and polymers, are used to produce lightweight and FRP composites with high mechanical properties [7,8,9]. GF is widely used as a reinforcing materials for FRP composites. The major advantages of GF are cheap, excellent in terms of tensile properties, resistance to chemical exposure and insulating properties. The disadvantages of GF are relatively high-density compared to other reinforcing fibers, high sensitivity to abrasion during handling, relatively low fatigue resistance, and high hardness that cause excessive wear on molding dies and cutting tools [10,11,12,13]. In addition to these synthetic fibers, plant fibers (also called natural fibers) are also used to reinforce polymers. In fact, they are now popular as viable alternatives to synthetic fibers, especially as a GF substitute in composite materials. Many studies have been conducted on hybrid natural fiber and GF [14,15,16,17,18,19]. Compared with other non-hybrid composite materials, the hybridization of GF and palm oil fiber leads to increased elongation at break and the highest impact strength [14,15,16]. Investigation on the mechanical properties of silk (biofiber) mixed with glass also showed that the performance of glass/biofiber FRP composites has been improved compared to non-hybrid composites [17]. Investigations subjecting bamboo-fiber-reinforced polypropylene (BFRP) and bamboo-GF-reinforced polypropylene hybrid (BGRP) composites to hysteretic tensile loading after hygrothermal aging by Thwe and Liao revealed that BGRP hybrids outperformed the BFRP composites in both fatigue resistance and retention of tensile stiffness and strength under environmental aging [18,19].

Generally, the higher performance of natural fiber-based FRP composites is achieved using fiber that has a higher content of cellulose and with more cellulose microfibrils aligned in the fiber direction [20,21]. These conditions tend to occur in bast fiber, which requires a higher structure to provide support for the stalk of the plant [14,22,23]. Besides fiber type, the interaction between the fiber and the polymer matrix must be considered to formulate natural fiber as reinforcement in polymer because it greatly affects the mechanical properties of polymer composites. The strength, stiffness and toughness of natural-fiber-reinforced polymer composites are hugely influenced by this factor.

An important aspect of the optimal mechanical properties of natural-fiber-reinforced polymer composites is to optimize the interface compatibility between the fiber and the polymer matrix [24]. Since the stress is transferred between the matrix and the fiber through the interface, good interface adhesion is required to obtain the best reinforcement effect. At the same time, natural fibers are extracted from different parts of the plant; these parts have different degrees of hydrophilicity, and the chemical properties are different from the matrix. Therefore, a proper bonding interface between the fibers is required to transfer effective stress from the matrix to the fibers and to perform effective bonding distribution across the interface. In addition, the interface is the key element responsible for the “reinforcement” in polymer matrix composites [25,26].

In general, physical and chemical interactions, chemical bonding, and mechanical interlocking can be used to explain the adhesion at the composite interface. To obtain extensive and appropriate interfacial bonding, good wetting between the fiber and the matrix is required [27,28,29,30]. For this purpose, the surface energy of both constituents plays an important role where the fiber should generally have higher surface energy than the liquid polymer resin. Besides facilitating good wetting, the surface energy preserves a stable contact after solidification of the composite [31]. In addition, the fiber–matrix interaction is also controlled by the functional groups on the surface of the fiber and the matrix in the interface contact zone. These functional groups determine the type of interface adhesion mechanism used [15,32,33].

The effective performance of the polymer matrix composites reinforced by natural fiber relies on the fiber-polymer matrix interface and its tendency of transferring stress to the fiber from the matrix [24,34]. Natural fiber inherently has poor compatibility with polymer matrix, high degree of moisture absorption and poor dimensional stability. All of these factors are the major hindrance to the perfect interfacial adhesion, which results in microcracking. This adversely affects the mechanical properties of the produced composites.

In addition, by selecting appropriate bonding system components and changing the surface topology of the fiber through appropriate treatment, the internal bonding force can be improved. A number of methods having various degrees of success are available for physical and chemical treatments of natural fiber to improve adhesion between fiber and polymer matrices [35].

One of the methods is physical methods, including corona, plasma, ultraviolet (UV), heat treatment and fiber beating. Physical treatment changes the structure and surface properties but does not widely change the chemical composition of the fiber [36,37]. In this way, the interface is usually strengthened by the mechanical bond between the fiber and the polymer [38]. However, to date, the chemical approaches are more signified within the literature rather than the physical approaches with better improvements [3,39,40].

On the other hand, chemical or pretreatment methods of the fiber chemically modify the surface of the fiber surface, reduce the moisture absorption and upsurge the surface unevenness [8,35]. The properties of cellulose, such as its hydrophilicity or hydrophobicity, elasticity, water absorption, adsorption or ion exchange capacity, resistance to microbial attack and heat resistance, can usually be modified through chemical treatments [41,42]. The main methods of cellulose chemical modification are esterification, etherification, halogenations, oxidation, and alkali treatment [43].

To date, the MMA-esterification of KBF has not been extensively studied. Previously, surface treatment on KBF with sodium hydroxide (NaOH) had been widely explored by researchers. However, MMA treatments on natural fibers were focused mainly on mechanical properties. Therefore, this study focuses on the adhesion of KFB interfacial adhesion of KBF and unsaturated polyester resin in composite systems, which can further contribute to the understanding of the improvement of mechanical properties. The initiative to optimize the potential of natural fiber as reinforce the material in polymer composite with the inclusion of GF was explored in this study. For this purpose, FTIR spectra and SEM micrographs of treated and untreated samples are carried out to determine the new functional group formed after treatment and to determine the adhesion between fibers to the matrix in composites. The performance of treated MMA-esterification kenaf composites was further enhanced with the addition of GF into the composite system. This can be used to reference the expansion technology of high-performance natural/synthetic-based hybrid composites.

## 2. Materials and Methods

### 2.1. Materials

Long KBF bundles used as reinforcements in this study were supplied by National Kenaf and Tobacco Board (NKTB), Kota Bahru, Kelantan, Malaysia. Electronic glass woven roving type GF (EWR500) is manufactured by Jiujiang Huirong New Material Co., Ltd., (Jiujiang, China) and provided by Hasrat Bestari Sdn. Bhd. Butterworth, Penang. Reversol P-9565 unsaturated polyester (UPE) resin was used as a polymer matrix in the composites fabrication. The resin is manufactured by Synthomer PLC and supplied by Dr. Rahmatullah Holding Sdn. Bhd., Bukit Mertajam, Penang, Malaysia. Butanox M-50, methyl ethyl ketone peroxide (MEKP), is manufactured by Akzo Nobel and supplied by Hasrat Bestari Sdn. Bhd. Butterworth, Penang, Malaysia. MEKP acts as a catalyst to initiate the crosslinking of UPE resins in the production of composite materials. Maleic anhydride (MA) was purchased from Sigma-Aldrich, Subang Jaya, Selangor. It was used as a chemical reagent for the esterification of KBF. N,N-Dimethylformamide (DMF) and hydroquinone were from Fisher Scientific and purchased from Dr. Rahmatullah Holding Sdn. Bhd., Bukit Mertajam, Penang, Malaysia.

### 2.2. Esterification of KBF with Maleic Anhydride

The long KBF was manually cut using a Heavy Duty Guillotine Paper Cutter (Dongguan Jiaxi Office Machine Co., Ltd. Dongguan, China), which has been set to cut the fiber to 10 mm length to ensure uniform length. Ethanol was added to the fiber and washed at room temperature for 3 h under constant stirring to remove organic soluble substances. The fibers were then filtered out, and the remaining solvent was removed by evaporation in an oven at 80 °C for 24 h. Maleic anhydride was diluted in N,N-dimethylformamide with a ratio of 3:7 (weight:weight). 5% of hydroquinone was added as an inhibitor in esterification treatment based on the weight of MA [44]. KBF with a ratio to the solvent being 1:20 (weight:weight) was put into the MA solution, making sure that all the KFB was fully covered with the solution and reacted at 90 °C in the reaction flask. The reaction was conducted with constant stirring for 60, 90 and 120 min. The esterified KBF was then filtered and rinsed with acetone. After this, treated fiber was refluxed with excess acetone for 3 h to eliminate unreacted MMA and placed in an oven at 80 °C for 24 h to dry. The weight percent gain (WPG) of the esterified KFB was calculated using Equation (1) [10,45]:

(1) $$\mathrm{WPG}\left(\%\right)=\frac{W_{b}{\;-\;W}_{a}}{W_{a}}\;\times \;100$$

where W_a_ is the weight of the fiber before the MA-treatment and W_b_ is the weight of the fiber after the MA-treatment.

### 2.3. Kenaf Mat Preparations

Long strand KBF was cut into 10 mm. The fiber mat was prepared manually using a deckle box with a nylon sieve mesh at the bottom for water drainage according to the mat preparation method reported by Rozman et al. [46]. Random orientation of KBF in the mat was obtained since the mat was manually prepared. Then the KBF mat was drained, removed from the deckle box and dried in the oven at a temperature of 80 °C for 24 h to remove excess water without degrading the KFB.

### 2.4. Composites Preparation

**Figure 1:**
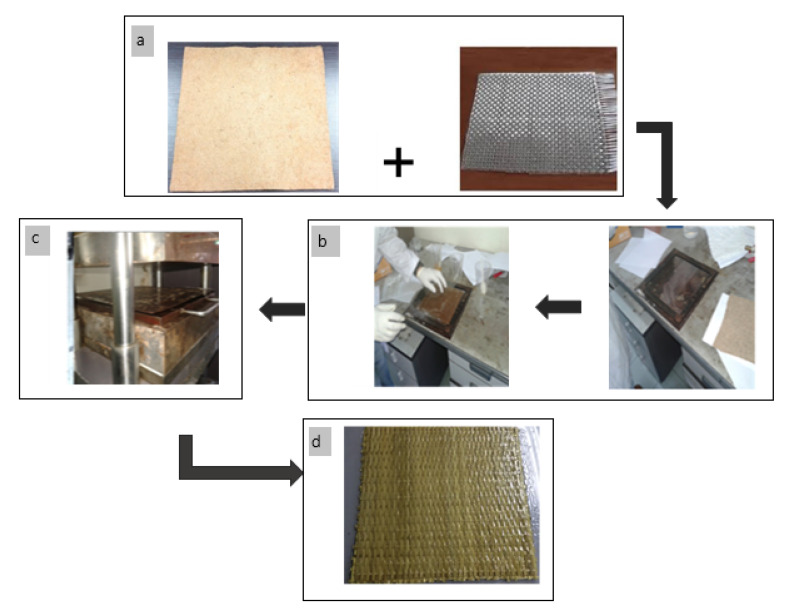
Fabrication method for KBF/GF hybrid composite; (**a**) KBF mat and GF mat; (**b**) placement of KBF at the stainless steel mold; (**c**) cold-pressed of the mold; (**d**) cured sample.

**Figure 2:**
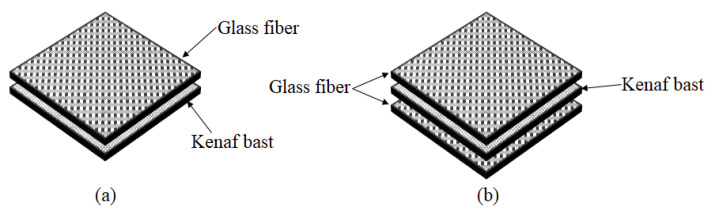
Sequence of (**a**) KBF + 5 wt % of GF and; (**b**) KBF + 10 wt % of GF for composite preparation.

The composite material is prepared in a stainless steel mold with a size of 200 mm × 200 mm × 5 mm by manual lay-up. KBF composites were prepared by placing the kenaf mat into stainless-steel mold and impregnated with UPE resin-based formulation, as shown in Table 1. Based on the UPE weight shown in Table 1 and Table 2, 2 wt % of MEKP was added to the UPE resin as a catalyst. The hybrid KBF/GF composites were prepared with the same method but with an addition of GF. The woven roving GF mat was cut into 150 mm × 150 mm to ensure that each layer of GF is equivalent to 5 wt %. Both kenaf and woven roving GF mats were alternately stacked and placed in a 150 mm × 150 mm × 5 mm stainless steel mold. The composite fabrication flow is shown in Figure 1, and the sequence of KBF with GF (5 wt %) and KBF with GF (10 wt %) for hybrid composite fabrication are shown in Figure 2. Fiber mats used in composite fabrication are shown in Figure 1a. The UPE resin and MEKP mixture were then poured onto the stacked fibers mat. A hand roller was used to ensure good resin impregnation and distribution into the fibers mat. The UPE impregnated KBF mat was placed in the stainless steel mold, as shown in Figure 1b with 5 mm thickness to achieve the constant thickness of the composite. Two stainless steel sheets were placed on top and at the bottom of the mold to cover the mold. The molding was then cold-pressed, as shown in Figure 1c, using a plastic hydraulic molding press, GT 714-P, Gotech Testing Machines, Inc, Taichung, Taiwan at 10 MPa to ensure good resin impregnation and eliminate void in the composite. Then, the pressed sample was left for 24 h to cure at room temperature. Composites with different fiber loadings and resins were prepared using the same methods. Figure 1d shows the cured composite sample.

## 3. Testing and Characterization

### 3.1. Tensile Test

**Figure 3:**
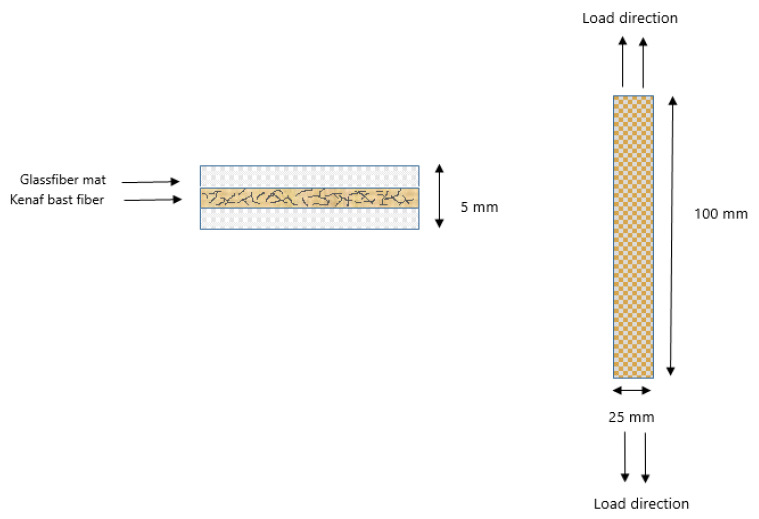
Tensile-load direction for KBF-treated MMA-esterification/ GF hybrid composite.

The composites were cut into dimensions of 100 mm × 25 mm × 5 mm for test specimens. The tensile test was conducted using the 3382A floor model universal testing system, US, according to ASTM D3039/ D3039M-17 [47]. Figure 3 shows the tensile load direction for the hybrid composite sample.

### 3.2. Scanning Electron Microscope (SEM)

A JEOL 6460LA scanning electron microscope (SEM) of Japan JOEL Company (Tokyo, Japan), was used to observe the microstructure of the fracture surface of composite materials through the tensile test. Both the treated and untreated KBF composite materials were observed. Before observation, the sample was coated with a layer of platinum.

### 3.3. Fourier-Transform Infrared (FTIR) Analysis

The functional groups of untreated and MA-treated KBF were identified using Fourier-transform infrared (FTIR) spectroscopy analytical techniques. Perkin Elmer Spectrum RX1 PC Ready, Perkin Elmer, Inc. US (Akron, OH, USA), operated at 4 cm^−1^ resolution, was used for the FTIR. The specimens were analyzed in the range of 4000–650 cm^−1^.

## 4. Results and Discussion

### 4.1. Reaction of Esterification Modification

**Figure 4:**
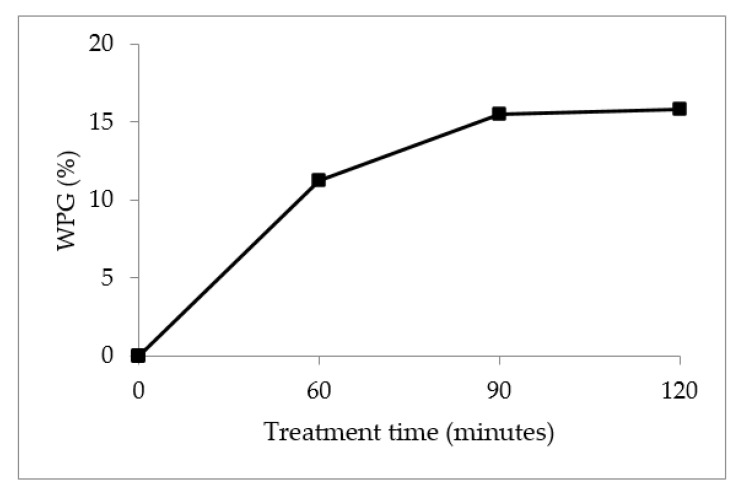
WPG of MA-treated KBF at different treatment times.

The chemical reaction will occur with the presence of the OH group from cellulose and hemicellulose in KBF. The functional relationship between the results of WPG and the esterification reaction of KBF with MA at 60, 90 and 120 min is shown in Figure 4. It is shown that with 60 and 90 min of treatment, WPG increases rapidly. Then the WPG remained constant for the 90 and 120 min treatment time. The fact that the MA and OH groups in the KBF reached the maximum reaction at 90 min can be explained. As a result, a minimal reaction occurs, and as the treatment time is extended, the weight increase of the fiber is suppressed. The increase in WPG value indicates that the KBF has been successfully esterified by MAA [48].

Before modification, DMF was used as a swelling agent and catalyst for KBF. The fiber cell wall swelled and exposed the OH groups on the surface of the cellulose, thereby allowing MA to react to form chemical bonds [44,49]. As the reaction time increases, more chemical bonds would have been formed between the OH groups and MA in cellulose. Time extension increases the reaction of free OH groups on the cellulose surface to react with MA and form chemical bonding.

**Figure 5:**
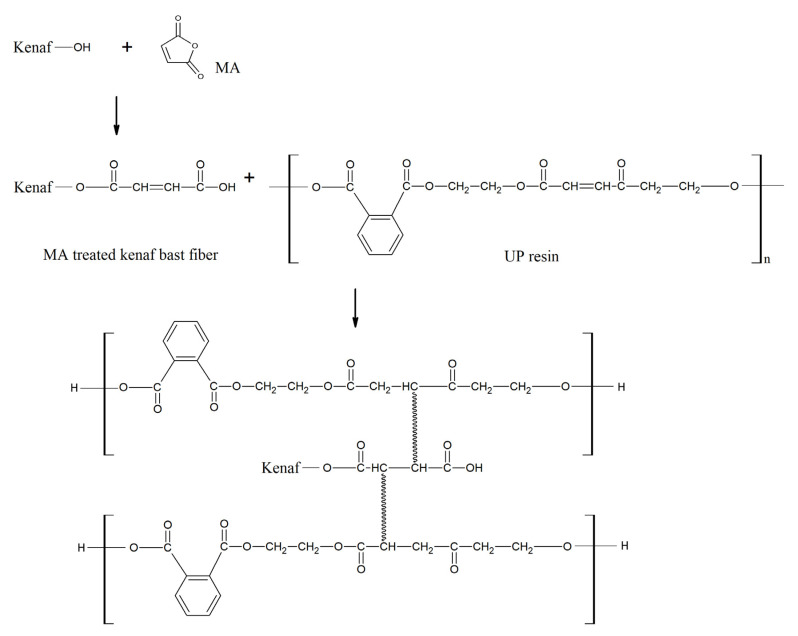
Reaction of MA-treated KBF and UPE resin.

Later, the modification improved the compatibility of modified KBF and UPE resin. It begins after the reaction between OH groups from the KBF and the anhydride groups of MA to form ester linkages, forming a covalent bond with the UPE matrix [50,51]. The appearance of the covalent bond is due to the ability of the C=C group in MA to copolymerize with the C=C site along the polyester chain through a free radical process [41], and the reaction is illustrated in Figure 5.

### 4.2. FTIR Analysis

**Figure 6:**
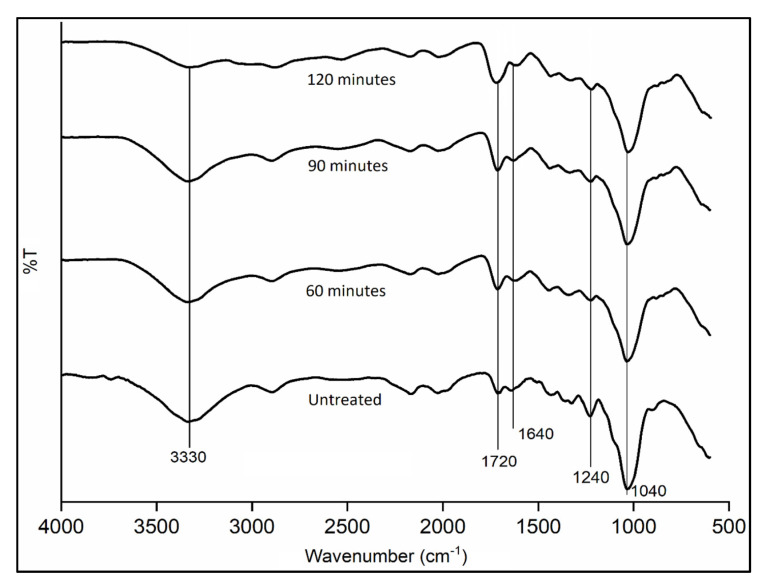
FTIR spectra for MA-treated KBF at different treatment time.

Figure 6 shows the reduction of OH content as the esterification treatment time increased. MA reacted with KBF cellulose and freed the OH groups from the hydrogen bonding, giving the differences at the major peak at around 3330 cm^−1^, corresponding to the O–H stretching vibration. The absorption peak at this region is observed to decrease.

The changes of the peak at about 1720 cm^−1,^ as shown in Figure 6 and Table 3, indicate the occurrence of esterification reaction. For the untreated KBF, the peak is associated with carbonyl C=O stretching of acetyl groups of hemicellulose. The absorption of KBF in this area with MA is greater, which signifies the increase of the C=O due to the attachment of ester group content. The peak in this region is attributed to the presence of an ester bond within the carboxylic group of the fiber. Whereas, as the treatment time increased, the absorption at this region increased significantly, which resembles the extent of esterification. The incremental appearance of the ester band provides evidence of the esterification of the OH group from KBF through anhydride modification. This finding is also in line with those by Khalil et al. [48] and Cantero et al. [52].

The increase in the absorption band at the 1640 cm^−1^ feature is due to the presence of C=C bonds, which is related to the maleate ester of MA that is attached to KBF after the treatment. At about 1240 cm^−1^, the absorption bands corresponded to the C–O stretching of the acetyl group and C=O stretching of the aryl group derived from the aromatic hydrocarbon ring of lignin. This is also in agreement with research findings by Jonoobi et al. [53]. As shown for all treatment times (60, 90 and 120 min), the intensity of the band decreased after the treatment. This could be due to the partial removal of lignin from the fiber surface. The peak appearing at around 1040 cm^−1^ is attributed to the C–C, C–OH and C–H stretching frequency of xylans and C–O–C stretching frequency belonging to the linkage of glycosidic, which is believed to be originated from hemicellulose in the fiber.

### 4.3. Tensile Properties

**Figure 7:**
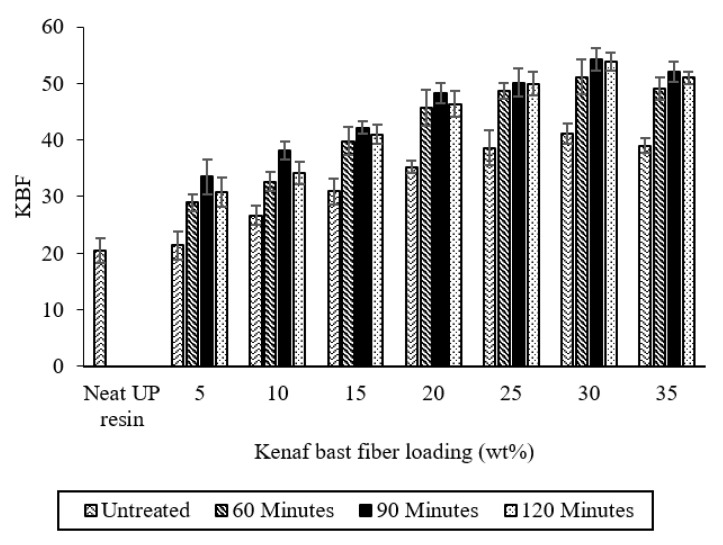
Effect of treatment time and KBF loading on tensile strength of UPE composites.

The effect of different treatment times and KBF content on tensile strength of untreated and MA-treated KBF-reinforced UPE composites is shown in Figure 7. It can be observed that the tensile strength increases as the treatment time increases, up to 90 min. As the treatment time increased (120 min), the tensile strength of the composite was slightly decreased. The addition of untreated and MA-treated KBF from 5 wt % to 30 wt % into the UP matrix had continuously increased the tensile strength of the composites.

The modification of KBF by MA increased the tensile strength, indicating the effectiveness of stress transfer from the UPE matrix and the KBF was enhanced. This is attributed to the increased compatibility due to the formation of a better bridging between the fiber and the matrix in the interface area [54,55]. It begins after the reaction between the OH group from KBF and the anhydride group from MA to form ester linkages, which in turn formed a covalent bond with the UPE matrix [50,51]. This occurrence of covalent bonding is due to the copolymerization of C=C groups of MA and C=C sites along the UPE chain through a radical process [41], and the reaction is illustrated in Figure 5. The result of the bonding has created a better and longer bridging linkage compared to shorter ester linkage of untreated KBF and UPE resin. A better and longer bridging linkage leads to the enhancement of compatibility between the reinforcement and matrix.

Tensile strength showed slight decrement as the treatment time is increased to 120 min. This could be due to the maximum reaction that was reached at 90 min, and the KBF was not significantly modified with further treatment time. This is also reflected in Figure 4, where there is no notable change in WPG at 90 min and 120 min treatment time. In addition, longer treatment times or excessive treatment may damage the fibers due to cellulose degradation and fiber cracking [14,16]. This situation will weaken the role of the fiber as a reinforcing material, resulting in a decrease in the mechanical properties of the composite material. The increment of KFB untreated and treated with MMA-esterification reduced the tensile strength of the composite. This is due to insufficient wetting of fiber by matrix. It is also believed that the increase of KFB loading to 30% and 35% contribute to poor distribution of KBF that subsequently promotes week points within the composite and lowers the tensile strength.

**Figure 8:**
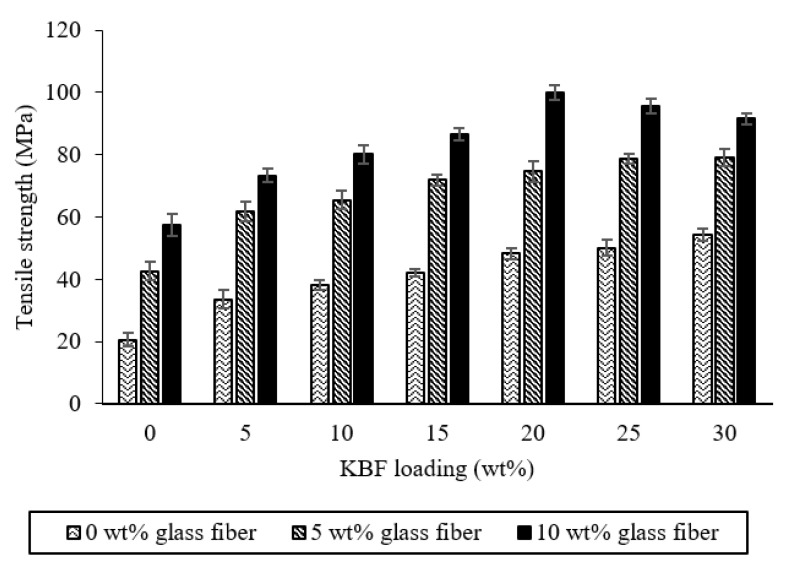
Effect of KBF and GF loading on tensile strength of hybrid MA-treated kenaf bast/GF-reinforced UPE composites.

The changes in tensile strength caused by the load of KBF and GF appeared in the KBF/GF hybrid composite system treated for 90 min. The performance of composite materials depends to a large extent on the performance of individual fibers. It can also be seen from Figure 8 that the hybrid composite material containing 10 wt % GF shows higher tensile strength compared to 5 wt % GF. For the hybrid composite composition containing 10 wt % GF, the composites were layered with GF on the outer layer on both sides of the reinforcing fiber. The tensile properties of GF have been known to be higher than natural fiber [14,56].

Furthermore, a good adhesion at the interface between the UPE matrix with GF and KBF has increased the load transfer efficiency between these three components. The establishment of good interfacial adhesion is due to the good compatibility of GF with the UPE matrix and the covalent bond formation of MA-treated KBF with the UPE matrix. This has led to the hybrid composites being able to withstand higher tension loads imposed. However, as the KFB loading increased after 20% with the addition of 5% and 10% GF into UPE composite, the tensile strength reduced due to insufficient matrix to wet the reinforcement materials.

### 4.4. SEM

**Figure 9:**
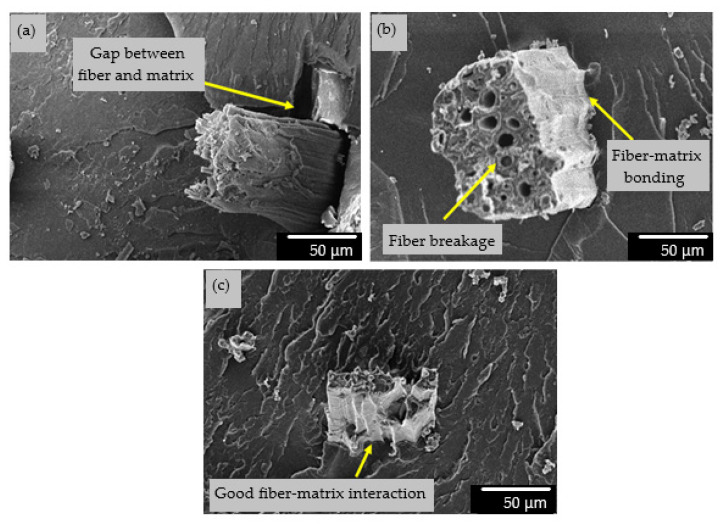
SEM micrographs KBF (20 wt %)-reinforced UPE composites tensile fracture surfaces at ×500 magnification. (**a**) Untreated KFB (**b**) KBF-treated 60 min MA (**c**) KBF-treated 90 min MA.

**Figure 10:**
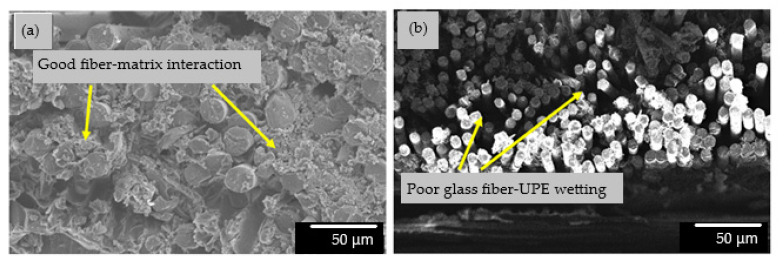
SEM micrographs KBF-treated MMA-esterification/GF hybrid composite at ×100 magnification. (**a**) Hybrid composite 20% KBF/10% GF content (**b**) hybrid composite with 30% KBF/10% GF content.

Figure 9 shows the SEM micrographs of untreated and MA-treated KBF-reinforced UP composites at 60 min and 90 min, respectively. Obviously, the KBF is firmly fixed by the UPE matrix, and there are signs of fiber fracture, which occurs during the failure of the composite material, as shown in Figure 9b,c. This indicates that the interaction between the fiber and the matrix occurred, which leads to good interfacial adhesion. For the untreated KBF-reinforced UP composites, the fiber is loosely embedded in the UPE matrix. The gap formed demonstrates poor adhesion between the fibers and the matrix can also be perceived in Figure 9a. This shows that the UPE resin has caused poor interaction on the untreated KBF. Without good interaction, strong interfacial bonds between the fibers and the matrix are unlikely to be achieved. Figure 10a illustrates the good wetting between reinforcing materials and matrix for hybrid composite with 20% KBF-treated MMA-esterification/10% GF. The increase of KBF-treated-MMA-esterification content/ 10% GF had reduced the wetting ability due to insufficient matrix polymer in polymer composite system, as shown in Figure 10b.

## 5. Conclusions

The effects of MMA-esterification treatment and fiber loadings (KBF and GF) on tensile strength and fiber-to-matrix interfacial-adhesion-reinforced UPE composites and hybrid composites were investigated. SEM was used to observe the tensile fracture surface and fiber morphologies. The functional group difference of MMA to KBF was also studied by FTIR. GFs were included in the kenaf bast treated MMA-esterification composite to further reinforce the hybrid composite. Based on the results obtained from the analysis and experimental works, the following conclusions can be drawn:

- The tensile strength of MMA-esterified KBF-reinforced UPE composites and hybrid composites increased with the increase of the content of KBF and GF;
- KFB treated with MMA showed a new functional group, and the increase of WPG as treatment time increases indicated the success of kenaf modification;
- Compared with untreated KBF-reinforced UPE composite material, MMA-esterified UPE composite material has a fiber braking effect instead of pulling out the fiber, and the gap between the fiber and the matrix is smaller (SEM micrographs);
- The addition of GFs to natural fiber composite systems shows better mechanical properties, thus indicating that hybrid composites are more suitable for use as final products.

In the future, it will be beneficial to expand this research to study the crosslinking between MA esterified KFB UPE composite and the treated hybrid composite to describe and evaluate crosslinking of treated MMA KFB with the polymer matrix. This will further prove the effectiveness of the MMA of natural-fiber-esterification treatment in polymer and hybrid polymer composites.

## Figures and Tables

**Table 1:** Composition of KBF-reinforced UPE composites.

KBF (wt %)	GF (wt %)	UPE (wt %)
0	0	100
5	0	95
10	0	90
15	0	85
20	0	80
25	0	75
30	0	70
35	0	65

**Table 2:** Composition of hybrid KBF/GF-reinforced UPE composites.

KBF (wt %)	GF (wt %)	UPE (wt %)
0	0	100
5	0	95
10	0	90
15	0	85
20	0	80
25	0	75
30	0	70
5	5	90
10	5	85
15	5	80
20	5	75
25	5	70
30	5	65
5	10	85
10	10	80
15	10	75
20	10	70
25	10	65
30	10	60

**Table 3:** FTIR analysis for MA-treated KBF at different treatment times.

No.	Untreated	60 min Treatment	90 min Treatment	120 min Treatment	Frequency Ranges (cm^−^^1^)	Functional Group
1	3326.78	3335.62	3331.48	3338.25	3330	OH– stretching band
2	1728.71	1716.62	1716.50	1721.26	1700–1650	C=C stretching band
3	1605.90	1635.74	1646.43	1637.27	1650–1725	C=O stretching bend
4	1239.43	1239.62	1231.37	1235.76	1200–1150	C–O stretching band
5	1040.67	1032.76	1029.07	1040.32	1050–1000	C–O stretching band

## Data Availability

Data sharing not applicable.

## References

[1] Saba N., Paridah M.T., Abdan K., Ibrahim N.A. (2016). Dynamic mechanical properties of oil palm nano filler/ kenaf/epoxy hybrid nanocomposites. Constr. Build. Mater..

[2] Mochane M.J., Mokhena T.C., Mokhothu T.H., Mtibe A., Sadiku E.R., Ray S.S., Ibrahim I.D., Daramola O.O. (2019). Recent progress on natural fiber hybrid composites for advanced applications: A review. Express Polym. Lett..

[3] Atmakuri A., Palevicius A., Siddabathula M., Vilkauskas A., Janusas G. (2020). Analysis of mechanical and wettability properties of natural fiber-reinforced epoxy hybrid composites. Polymers..

[4] Manikandan V., Velmurugan R., Ponnambalam S.G., Thomas S. (2004). Mechanical properties of short and uni-directional aligned palmyra fiber reinforced polyester composite. Int. J. Plast. Technol..

[5] Velmurugan R., Manikandan V. (2007). Mechanical properties of palmyra/glass fiber hybrid composites. Compos. Part A Appl. Sci. Manuf..

[6] Jarukumjorn K., Suppakarn N. (2009). Effect of glass fiber hybridization on properties of sisal fiber–polypropylene composites. Compos. B Eng..

[7] Sonnenschein R., Gajdosova K., Holly I. (2016). FRP composites and their using in the construction of bridges. Procedia Eng..

[8] Wu C., Li V.C. (2017). Thermal-mechanical behaviors of CFRP-ECC hybrid under elevated temperatures. Compos. B Eng..

[9] Danraka M.N., Mahmod H.M., Oluwatosin O.K.J., Student P. (2017). Strengthening of reinforced concrete beams using FRP technique: A review. Int. J. Eng. Sci..

[10] Gunaslan S.E., Karaşin A., Oncu M.E. (2014). Properties of FRP materials for strengthening. Int. J. Innov. Sci. Eng. Technol..

[11] Ahmed A., Guo S., Zhang Z., Shi C., Zhu D. (2020). A review on durability of fiber reinforced polymer (FRP) bars reinforced seawater sea sand concrete. Constr. Build. Mater..

[12] Arthanarieswaran V.P., Kumaravel A., Kathirselvam M. (2014). Evaluation of mechanical properties of banana and sisal fiber reinforced epoxy composites: Influence of glass fiber hybridization. Mater. Des..

[13] Samanta S., Muralidhar M., Signh T.J., Sarkar S. (2015). Characterization of mechanical properties of hybrid bamboo/GFRP and jute/GFRP composites. Mater. Today.

[14] Pickering K.L., Efendy M.A., Le T.M. (2016). A review of recent developments in natural fibre composites and their mechanical performance. Compos. Part A Appl. Sci. Manuf..

[15] Tran L.Q.N., Fuentes C.A., Dupont-Gillain C., Van Vuure A.W., Verpoest I. (2013). Understanding the interfacial compatibility and adhesion of natural coir fibre thermoplastic composites. Compos. Sci. Technol..

[16] Edeerozey A.M., Akil H.M., Azhar A., Ariffin M.Z. (2007). Chemical modification of kenaf fibers. Mater. Lett..

[17] Sreekala M., George J., Kumaran M., Thomas S. (2002). The mechanical performance of hybrid phenol-formaldehyde-based composites reinforced with glass and oil palm fibres. Compos. Sci. Tech..

[18] Thwe M.M., Liao K. (2002). Effects of environmental aging on the mechanical properties of bamboo–glass fiber reinforced polymer matrix hybrid composites. Compos. A Appl. Sci. Manuf..

[19] Thwe M.M., Liao K. (2003). Durability of bamboo-glass fiber reinforced polymer matrix hybrid composites. Compos. Sci. Tech..

[20] Anwar Z., Gulfraz M., Irshad M. (2014). Agro-industrial lignocellulosic biomass a key to unlock the future bio-energy: A brief review. J. Radiat. Res. Appl..

[21] Mohammed L., Ansari M.N., Pua G., Jawaid M., Islam M.S. (2015). A Review on Natural Fiber Reinforced Polymer Composite and Its Applications. Int. J. Polym. Sci..

[22] Jones D., Ormondroyd G.O., Curlin S.F., Popescu C.M., Popescu C., Mizi D., Peng F. (2017). Chemical compositions of natural fibres. Advanced High Strength Natural Fibre Composites in Construction.

[23] Lee C.H., Khalina A., Lee S.H., Ming L. (2020). A Comprehensive Review on Bast Fibre Retting Process for Optimal Performance in Fibre-Reinforced Polymer Composites. Adv. Mater. Sci..

[24] Rajak D.K., Pagar D.D., Menezes P.L., Linul E. (2019). Fiber-reinforced polymer composites: Manufacturing, properties, and applications. Polymers.

[25] Xu Y., Kawata S., Hosoi K., Kawai T., Kuroda S. (2009). Thermomechanical properties of the silanized-kenaf/polystyrene composites. Express Polym. Lett..

[26] Senthilraja R., Sarala R., Antony A.G. (2020). Effect of acetylation technique on mechanical behavior and durability of palm fibre vinyl-ester composites. Mater. Today..

[27] Zhou Y., Fan M., Chen L. (2016). Interface and bonding mechanisms of plant fibre composites: An overview. Compos. B Eng..

[28] Fuentes C.A., Brughmans G., Tran L.Q.N., Dupont-Gillain C., Verpoest I., Van Vuure A.W. (2015). Mechanical behaviour and practical adhesion at a bamboo composite interface: Physical adhesion and mechanical interlocking. Compos. Sci. Technol..

[29] Popita G.E., Rosu C., Manciula D., Corbu O., Popovici A., Nemes O., Sandu A.V., Proorocu M., Dan S.B. (2016). Industrial Tanned Leather Waste Embedded in Modern Composite Materials. Mater. Plast..

[30] Kasa S.N., Omar M.F., Abdullah M.M.A., Ismail I.N., Ting S.S., Vac S.C., Vizureanu P. (2017). Effect of Unmodified and Modified Nanocrystalline Cellulose Reinforced Polylactic Acid (PLA) Polymer Prepared by Solvent Casting Method Morphology, mechanical and thermal properties. Mater. Plast..

[31] Zaghi A.E. (2018). Mechanical characteristics of hybrid composites with±45° glass and 0°/90° stainless steel fibers. Materials.

[32] Beter J., Maroh B., Schrittesser B., Mühlbacher I., Griesser T., Schlögl S., Fuchs P.F., Pinter G. (2021). Tailored Interfaces in Fiber-Reinforced Elastomers: A Surface Treatment Study on Optimized Load Coupling via the Modified Fiber Bundle Debond Technique. Polymers.

[33] Ferreira F.V., Dufresne A., Pinheiro I.F., Souza D.H.S., Gouveia R.F., Mei L.H.I., Lona L.M.F. (2018). How do cellulose nanocrystals affect the overall properties of biodegradable polymer nanocomposites: A comprehensive review. Eur. Polym. J..

[34] Li M., Pu Y., Thomas V.M., Yoo C.G., Ozcan S., Deng Y., Nelson K., Ragauskas A.J. (2020). Recent advancements of plant-based natural fiber–reinforced composites and their applications. Compos. B Eng..

[35] Kabir M., Wang H., Lau K., Cardona F. (2013). Effects of chemical treatments on hemp fibre structure. Appl. Surf. Sci..

[36] Husin M.M., Mustapa M.S., Wahab M., Tajul Arifin A.M., Ganasan R.A., Jais F.H. (2017). Characteristics on treated kenaf fiber reinforced polypropylene composites. Mater. Sci. Forum..

[37] Darus S.A.A.Z.M., Ghazali M.J., Azhari C.H., Zulkifli R., Shamsuri A.A., Sarac H., Mustafa M.T. (2020). Physicochemical and thermal properties of lignocellulosic fiber from Gigantochloa Scortechinii bamboo: Effect of steam explosion treatment. Fiber Polym..

[38] Priya S.P., Rai S. (2006). Mechanical performance of biofiber/glass-reinforced epoxy hybrid composites. J. Ind. Text..

[39] Li X., Tabil L.G., Panigrahi S. (2007). Chemical treatments of natural fiber for use in natural fiber-reinforced composites: A review. J. Polym. Environ..

[40] Li Z., Zhou W., Yang L., Chen P., Yan C., Cai C., Li H., Li L., Shi Y. (2019). Glass fiber-reinforced phenol formaldehyde resin-based electrical insulating composites fabricated by selective laser sintering. Polymers.

[41] Bessadok A., Roudesli S., Marais S., Follain N., Lebrun L. (2009). Alfa fibres for unsaturated polyester composites reinforcement: Effects of chemical treatments on mechanical and permeation properties. Compos. Part A Appl. Sci. Manuf..

[42] Hokkanen S., Bhatnagar A., Sillanpää M. (2016). A review on modification methods to cellulose-based adsorbents to improve adsorption capacity. Water Res..

[43] BorhanaOmran A.A., Mohammed A.A.B.A., Sapuan S.M., Ilyas R.A., Asyraf M.R.M., Rahimian Koloor S.S., Petr M. (2021). Micro and nanocellulose in polymer composite materials: A Review. Polymers.

[44] Rozman H.D., Musa L., Abubakar A. (2005). Rice husk–polyester composites: The effect of chemical modification of rice husk on the mechanical and dimensional stability properties. J. Appl. Polym. Sci..

[45] Musa L., Rozyanty A.R., Zhafer S.F. (2018). Effect of modification time of kenaf bast fiber with maleic anhydride on tensile properties of kenaf-glass hybrid fiber unsaturated polyester composites. Solid State Phenom..

[46] Rozman H., Rozyanty A.R., Tay G., Kumar R. (2010). The effect of glycidyl methacrylate treatment of empty fruit bunch (EFB) on the properties of ultra-violet radiation cured EFB-unsaturated polyester composite. J. Appl. Polym. Sci..

[47] American Society for Testing and Materials (ASTM) (2017). 2008 Standard Test Method for Tensile Properties of Polymer Matrix Composite Materials.

[48] Khalil H.A., Suraya N.L. (2011). Anhydride modification of cultivated kenaf bast fibers: Morphological, spectroscopic and thermal studies. Bioresources.

[49] Akhtar M.N., Sulong A.B., Nazir M.S., Majeed K., Radzi M.K.F., Ismail N.F., Raza M.R., Mohammad J., Salit M.S., Othman Y.A. (2017). Kenaf-biocomposites: Manufacturing, characterization, and applications. Green Biocomposites.

[50] Hong C., Kim N., Kang S., Nah C., Lee Y.S., Cho B.H., Ahn J.H. (2008). Mechanical properties of maleic anhydride treated jute fibre/polypropylene composites. Plast. Rubber Compos..

[51] Li K., Qiu R., Liu W. (2015). Improvement of interfacial adhesion in natural plant fiber-reinforced unsaturated polyester composites: A critical review. Rev. Adhes. Adhes..

[52] Cantero G., Arbelaiz A., Llano-Ponte R., Mondragon I. (2003). Effects of fibre treatment on wettability and mechanical behaviour of flax/polypropylene composites. Compos. Sci. Technol..

[53] Jonoobi M., Harun J., Mishra M., Oksman K. (2009). Chemical composition, crystallinity and thermal degradation of bleached and unbleached kenaf bast (*Hibiscus cannabinus*) pulp and nanofiber. Bioresources.

[54] Pradipta R., Mardiyati S., Purnomo I. (2017). Effect of maleic anhydride treatment on the mechanical properties of sansevieria fiber/vinyl ester composites. AIP Conf. Proc..

[55] Rahman R., Putra S.Z.F.S., Jawaid M., Thariq M., Saba N. (2019). Tensile properties of natural and synthetic fiber-reinforced polymer composites. Mechanical and Physical Testing of Biocomposites, Fibre-Reinforced Composites and Hybrid Composites.

[56] Srinivasan V., Boopathy S.R., Ramnath B.V. (2015). Investigation of flexural property of kenaf–flax hybrid composite. ARPN J. Eng. Appl. Sci..

